# InGaN/GaN multilayer quantum dots yellow-green light-emitting diode with optimized GaN barriers

**DOI:** 10.1186/1556-276X-7-617

**Published:** 2012-11-07

**Authors:** Wenbin Lv, Lai Wang, Jiaxing Wang, Zhibiao Hao, Yi Luo

**Affiliations:** 1 Tsinghua National Laboratory for Information Science and Technology, Department of Electronic Engineering, Tsinghua University, Beijing, 100084, People’s Republic of China; 2 Present Address: Tsinghua University, Room 2-305, Rohm Building, Beijing, 100084, People’s Republic of China

**Keywords:** InGaN quantum dots, GaN barriers, metalorganic vapor phase epitaxy, light-emitting diodes

## Abstract

InGaN/GaN multilayer quantum dot (QD) structure is a potential type of active regions for yellow-green light-emitting diodes (LEDs). The surface morphologies and crystalline quality of GaN barriers are critical to the uniformity of InGaN QD layers. While GaN barriers were grown in multi-QD layers, we used improved growth parameters by increasing the growth temperature and switching the carrier gas from N_2_ to H_2_ in the metal organic vapor phase epitaxy. As a result, a 10-layer InGaN/GaN QD LED is demonstrated successfully. The transmission electron microscopy image shows the uniform multilayer InGaN QDs clearly. As the injection current increases from 5 to 50 mA, the electroluminescence peak wavelength shifts from 574 to 537 nm.

## Background

In recent years, white light-emitting diodes (LEDs) have attracted much attention for applications in general lighting and liquid crystal display back-lighting [1,2]. White LEDs based on red, green, and blue LED chips exhibit higher color rendering index and higher luminous efficiency limit simultaneously, which is theoretically superior to the solution of blue LED plus yellow phosphor [3]. However, the performance of RGB white LEDs fails to meet expectation due to the well-known ‘green gap’ [4-6]. Additionally, in the spectral range of green gap, the efficiency of yellow-green LEDs decreases dramatically compared to that of short-wavelength green LEDs [7]. This is attributed to the low internal quantum efficiency (IQE) of InGaN/GaN multi-quantum wells (MQWs) with high indium composition. The dislocations and defects induced by the large lattice mismatch between InGaN and GaN act as nonradiative recombination centers, thus weakening the IQE [8]. Furthermore, the quantum-confined Stark effect (QCSE) of high-In-content InGaN/GaN MQWs leads to energy band tilting, which decreases the overlap integral of electrons and holes by spatial separation [9-12]. To circumvent the above disadvantages, various approaches are adopted, such as InGaN quantum dots (QDs) grown as the alternative active region [13,14] and InGaN QWs grown on nonpolar or semipolar planes [6,15]. In previous reports, growth behaviors of high-In-content InGaN quantum dots using a growth interruption method are intensively investigated [16-20], which paves the way to high-efficiency QD LEDs. As the capping layer of InGaN QDs, GaN barrier is critical to the performance of multilayer InGaN QDs. Previous literature indicates that GaN barrier exhibits a rough surface when grown under the same parameters as the InGaN QDs, which will influence the QD formation and distribution of different layers [21]. Thus, it is necessary to optimize the growth parameters of the GaN barrier layer in multilayer InGaN/GaN QD structure. Some GaN barrier growth approaches are believed to improve the structural properties of InGaN/GaN QD or QW structure, such as the growth of GaN barrier at higher temperature [22,23], using H_2_ as carrier gas in GaN barrier growth [22,24,25], and growth interruption after QW growth [24,26]. In this study, samples of InGaN QDs with GaN barrier layer structure have been grown by metal organic vapor phase epitaxy (MOVPE) with different growth parameters. It is shown that the surface morphology of the GaN barrier layer is improved by increasing the growth temperature and switching the carrier gas from N_2_ to H_2_. Based on the optimized growth parameters, a yellow-green LED including 10-layer InGaN/GaN QDs is successfully fabricated. Transmission electron microscopy (TEM) image indicates a uniform multilayer InGaN QD structure clearly. The electroluminescence peak of the LED shows a blueshift from 574 to 537 nm as the current increases from 5 to 50 mA.

## Methods

**Figure 1:**
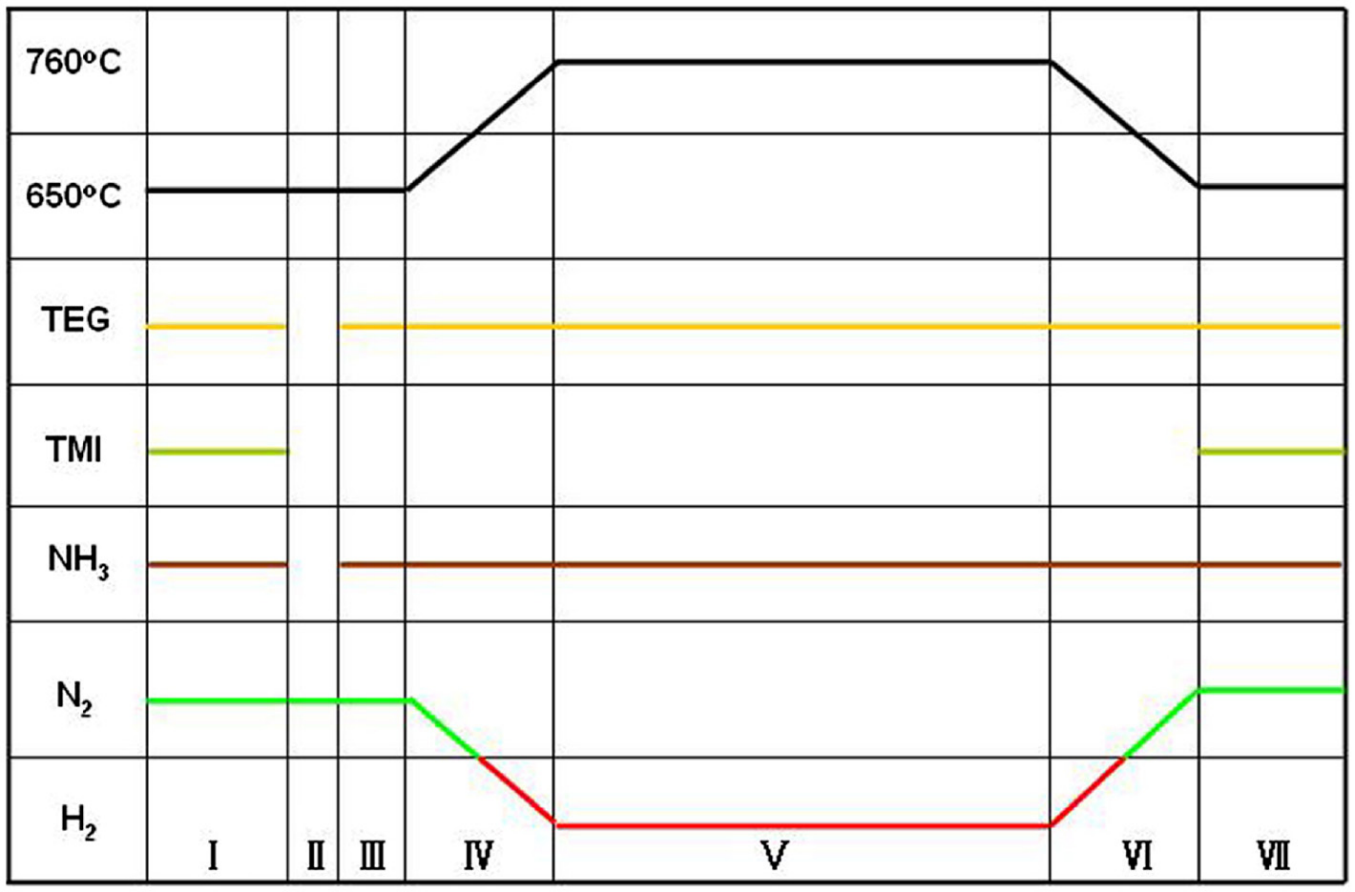
**Parameters used in growing a single InGaN QD and GaN barrier period in sample E.** Regions indicating the different growth periods: I, InGaN QD; II, growth interruption; III, protective GaN barrier; IV, temperature ramping and gas switching; V, GaN barrier; VI, temperature decreasing and gas switching; VII, InGaN QD.

All samples in this study were grown on (0001) sapphire substrates in an AIXTRON 2000HT MOVPE system. Four samples, labeled as A, B, C, and D, were prepared to investigate the growth parameters of GaN barrier layers. For sample A, the substrate was thermally cleaned by H_2_ gas at 1,060°C for 10 min as usual. After that, a 30-nm GaN buffer layer was deposited at 530°C followed by a 2-μm GaN bulk layer grown at 1,040°C. The reactor temperature was then decreased to 650°C, and N_2_ was used as the carrier gas in place of H_2_ for InGaN QD growth. A single layer of InGaN QDs was grown by the growth interruption method [17,19,20], which included the first nominal 1.5-nm InGaN film growth and the following 20-s growth interruption. The flow rates of triethylgallium and trimethylindium were 1.69 × 10^−5^ mol/min and 2.07 × 10^−5^ mol/min, respectively, while the flow rate of NH_3_ was 0.625 mol/min with nitrogen as the carrier gas, corresponding to a V/III ratio of approximately 16,600. Compared with sample A, samples B, C, and D all added a 30-nm GaN capping layer, but they were distinguished by the growth parameters, which were listed in Table 1. During the GaN barrier layer growth process of sample B, the carrier gas and temperature are kept the same as those for the growth of InGaN QDs. While in the first 1 min of the GaN barrier growth of sample C, the same carrier gas and temperature as in the InGaN QD growth were used in order to maintain the QD structure. Then, the carrier gas is switched to H_2_. Also, sample D not only switches the carrier gas to H_2_ and but also increases the growth temperature to 760°C in 1 min and a half. Using the growth parameters of sample D, a yellow-green LED including 10-layer InGaN/GaN QDs as the active region was grown, which was labeled as sample E. The active region was sandwiched between a 4-μm n-GaN bulk layer and the p-AlGaN (30 nm)/p-GaN (160 nm) layers. Figure 1 shows the whole profile of temperature changing and carrier gas switching in the growth process. During the whole growth process of the GaN barrier, only the In precursor was shut off, while the same fluxes as in the growth of InGaN QDs were maintained for the Ga and N precursors. To compare the optimized growth parameters, sample F was grown with a five-layer InGaN/GaN QD structure in which the GaN capping layer was grown at the InGaN growth temperature with nitrogen as ambient.

**Table 1:** Growth parameters of samples B, C, and D

**Sample**	**GaN barrier growth temperature (°C)**	**GaN barrier growth ambient**
B	650	N_2_
C	650	H_2_
D	760	H_2_

The surface morphologies of samples A, B, C, and D were measured by PSIA X-100 atomic force microscope (AFM). The QD density and average diameter and height were calculated by the Scanning Probe Image Processor software. The cross section of sample E was observed at 200 kV by Tecnai G20 TEM system. For Z-contrast imaging of sample F, JEOL-2010F equipped with a high-angle annular dark-field detector for scanning TEM was used. The photoluminescence (PL) of samples B, C, D, and E was measured using a 325-nm He-Cd laser as the excitation source. The excitation light spot diameter was 1 mm, and the excitation optical power was 27 mW. Sample E was then processed into 300 × 300-μm^2^ chips. The n-GaN is exposed by etching with inductively coupled Cl_2_/BCl_3_/Ar_2_ plasma. Ni/Au was deposited on the transparent electrodes, and the transparent electrodes were annealed at 600°C for 3 min in oxygen ambient. Cr/Au was deposited on the n-GaN layer and p-GaN layer as n- and p-electrodes.

## Results and discussion

**Figure 2:**
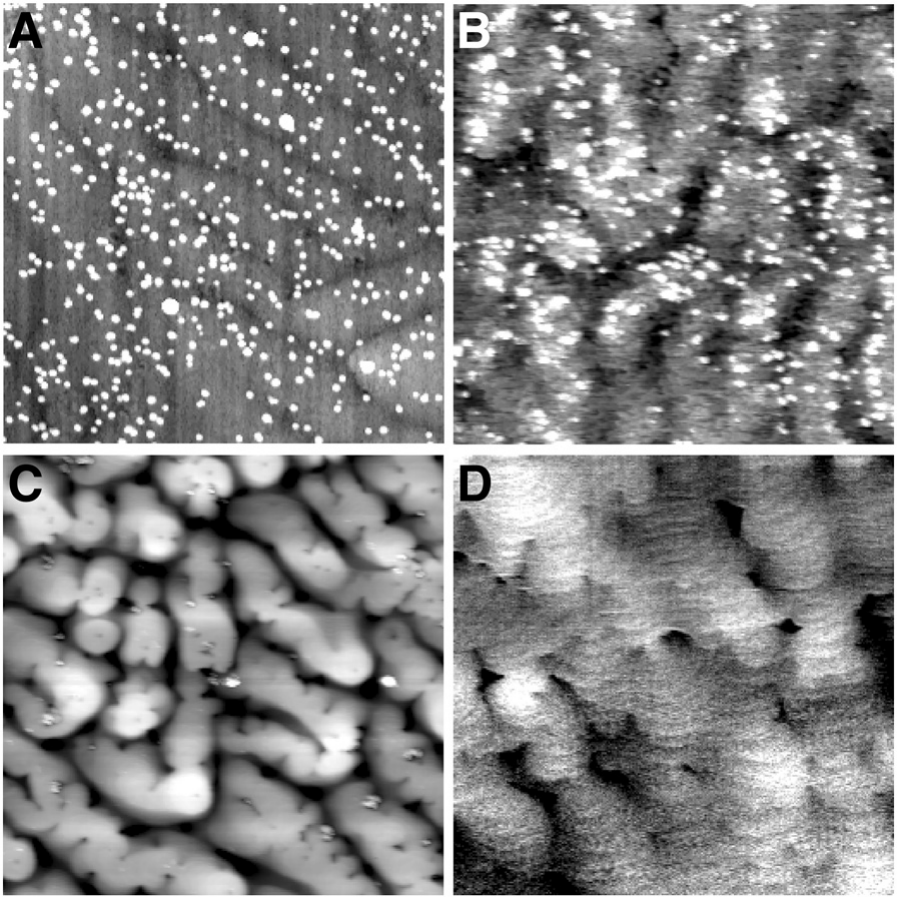
**AFM images (a, b, c, d) of samples A, B, C, and D (5 × 5 μm**^**2**^**).** The root mean square roughness of samples B, C, and D are 1.937, 1.815, and 0.416 nm, respectively.

**Figure 3:**
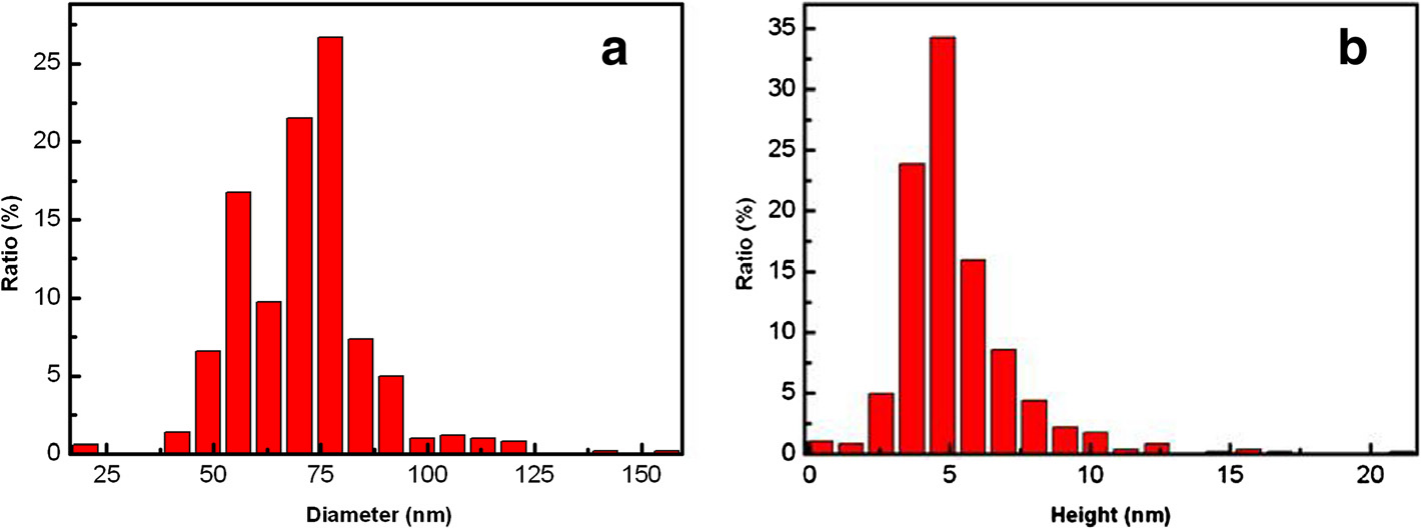
Diameter (a) and height (b) distribution histogram of sample A.

The AFM images of samples A, B, C, and D are shown in Figure 2. The InGaN QDs can be observed clearly in sample A. The diameter and height distribution histogram of sample A are depicted in Figure 3a,b, and the diameter, height, and density statistics are 73.5 nm, 5.1 nm, and 2.0 × 10^9^ cm^−2^. However, when a GaN capping layer was stacked on sample A, the surface exhibits a different appearance under different growth parameters. The surface of sample B shows hillock shapes. This is attributed to the unoptimized growth parameters. When GaN barrier is grown at 650°C, the Ga atoms might not have enough energy to migrate to the proper area at low temperature [27,28]. Using N_2_ instead of H_2_ as carrier gas, the density of gas molecules increased near the reaction surface, which leads to decreased surface diffusion length of the reactants [29]. By decreasing the flow rate of N_2_ and increasing that of H_2_, the density of gas molecules decreased near the reaction surface, which leads to increased surface diffusion length of the reactants [29]. Thus, the surface morphology of sample C is improved compared to that of sample B. Furthermore, by increasing the growth temperature, the morphology of sample D becomes much smoother. This is because Ga atoms might have enough energy for migration as the temperature increases [27,28]. The root mean roughness decreases from samples B to D in Figure 2b,c,d, which confirms the above analysis.

**Figure 4:**
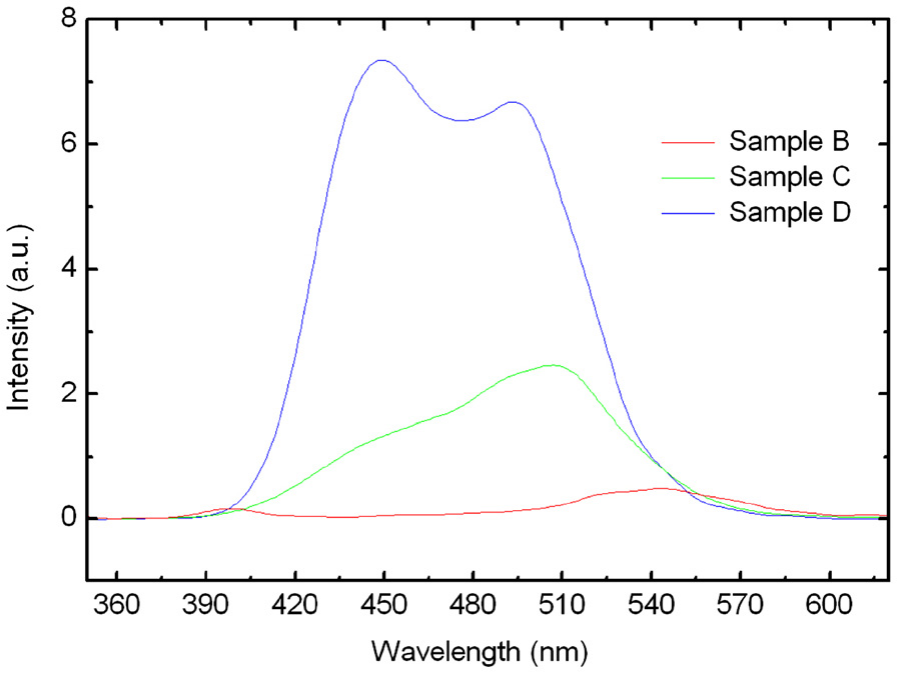
PL spectra of samples B, C, and D at room temperature.

The room-temperature PL spectra of samples B, C, and D are shown in Figure 4. Sample D has the highest PL intensity among the three samples, while sample B has a rather weak PL intensity. It confirms the improved crystalline quality when using H_2_ as ambient and higher growth temperature for the GaN barrier. In many literatures, the etching effect of In content has been reported when using H_2_ as carrier gas in the reactor [29,30]. H_2_ is known to eliminate the In-rich clusters in the InGaN QD/GaN barrier interface, thus decreasing the average In content. The PL peak wavelength shows blueshift from sample B to sample D, which confirms the H_2_ effect in the GaN barrier growth. Due to the lattice mismatch between InGaN and GaN, the decrease of In-rich clusters will benefit the crystalline quality of the GaN barrier layer. Thus, the crystalline quality is improved with changing the carrier gas from nitrogen to hydrogen during GaN barrier layer growth, which confirms the result of the previous reports [22].

**Figure 5:**
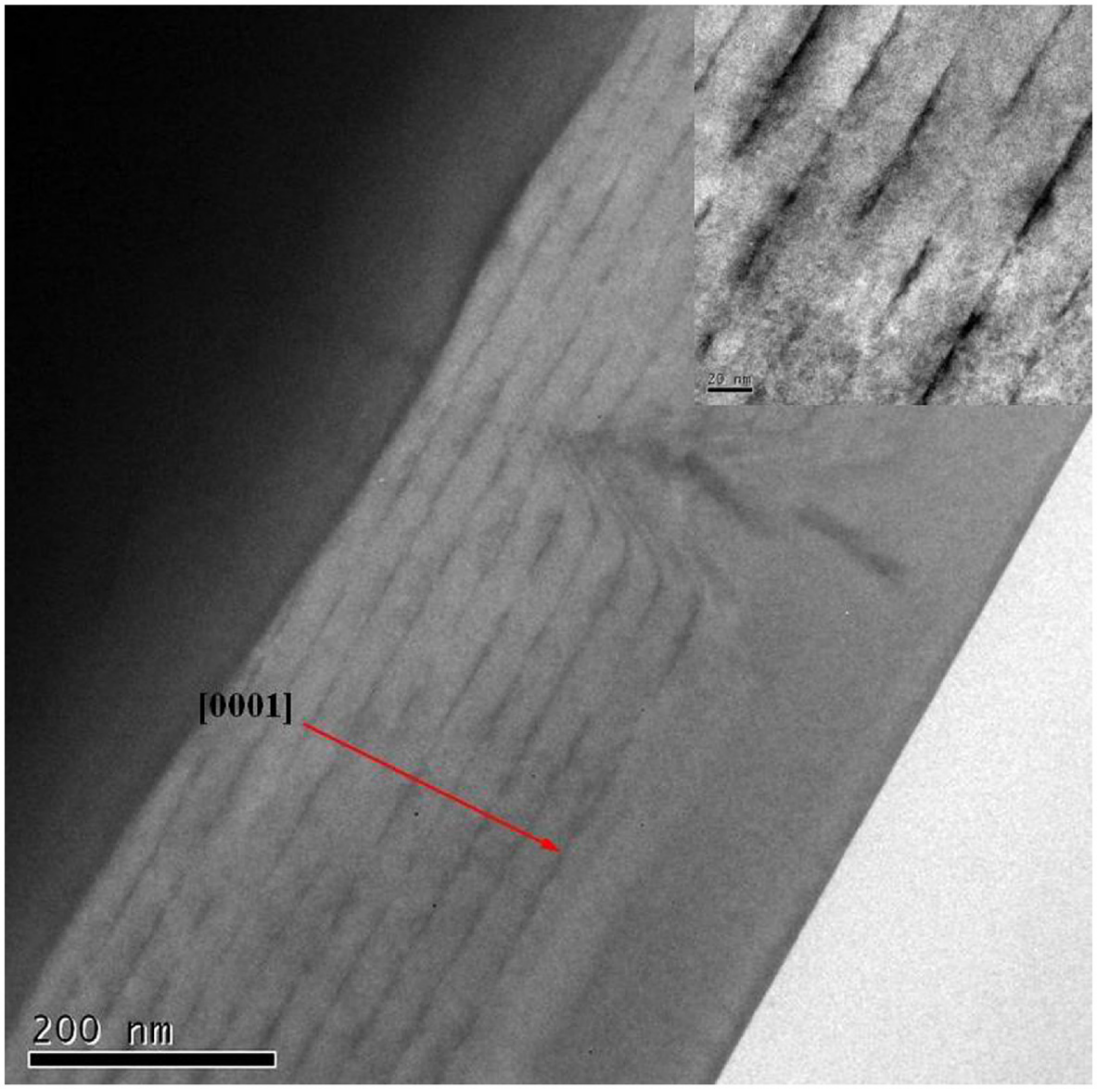
**Cross-sectional TEM image of samples E.** The inset shows the details of the multilayer InGaN/GaN structure.

The cross-sectional TEM image of sample E is shown in Figure 5. The 10-layer uniform QDs and the flat surface of the GaN barrier layer can be observed clearly. This result validates the growth method mentioned above. The average QD diameter and height of sample A and sample E are listed in Table 2. These two samples correspond to the single-layer and multilayer InGaN QDs. It proves that InGaN QDs maintain the three-dimensional nanostructure during the multilayer growth. The average diameter and height for the 10-layer QD structure is smaller to some extent than those of the single-layer QD sample. H_2_ ambient and elevated temperature induce partial decomposition of InGaN [30,31], which is more pronounced for the 10-layer QD sample than the single-layer one. Meanwhile, the oblique angle of the TEM image should be taken into account, which may cause the average diameter and height to be distorted to some extent. Considering the above factors, these data are consistent with those of single QD layer measured with AFM.

**Table 2:** The comparison of QD diameter and height between samples A and E

	**Sample A**	**Sample E**
Diameter (nm)	73.5	57.3
Height (nm)	5.1	4.1

**Figure 6:**
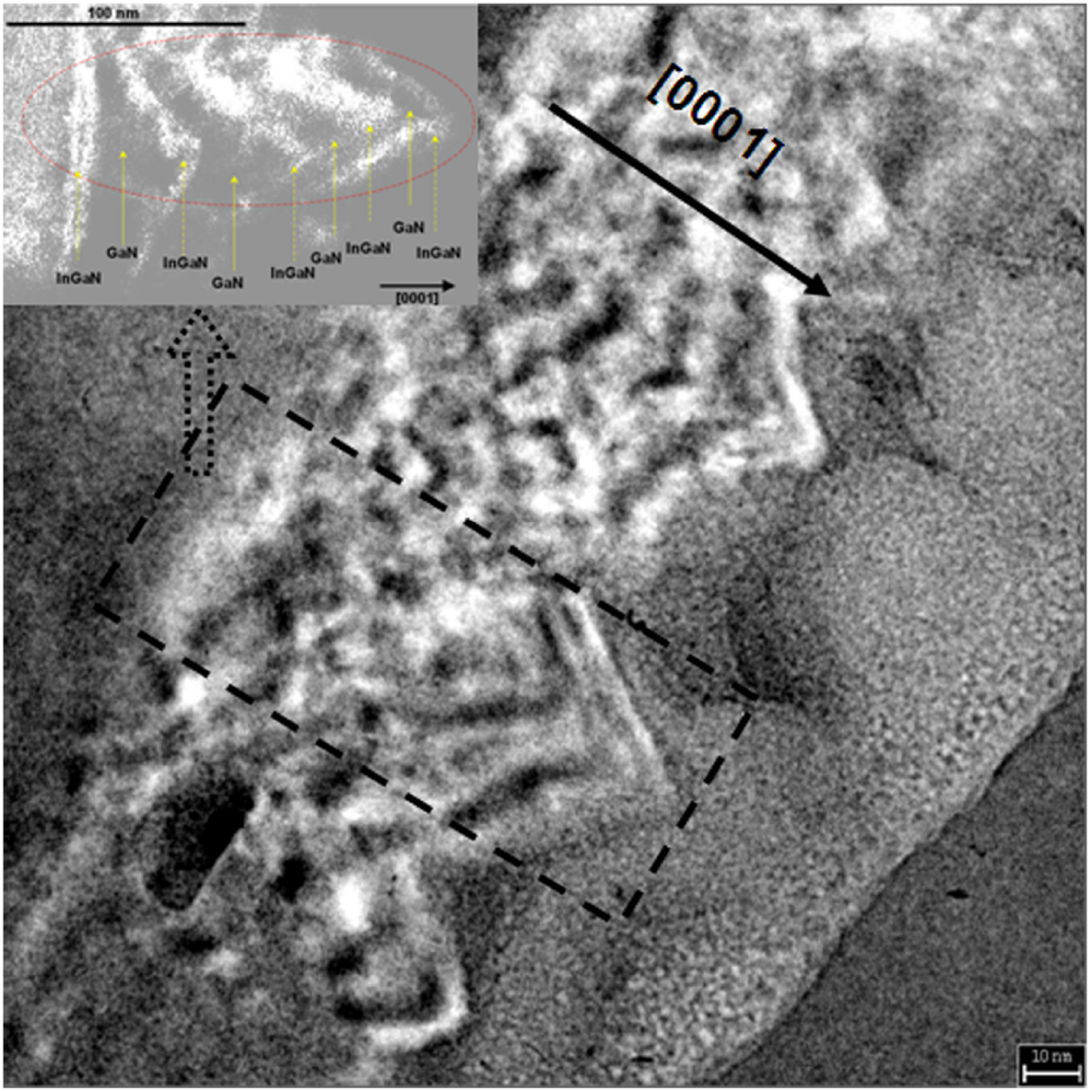
**Cross-section TEM image of samples F.** The inset shows the details of the multilayer InGaN/GaN structure.

The GaN barrier layer of sample F was grown at the InGaN growth temperature and using nitrogen as carrier gas. Z-contrast imaging is known to be sensitive to composition, which indicates that the brighter regions respond to the In-rich regions. Thus, the cross-section TEM image of sample F indicates that the surface morphology of the GaN barrier layer is rather rough in Figure 6. It will influence the uniformity and morphology of InGaN QDs in different layers. On the other hand, the GaN barrier layer of sample E was grown at higher temperature and using hydrogen as carrier gas. According to the TEM image, the sizes of the QDs in different layers were fairly uniform. Compared with sample F, based on the flat GaN barrier layer grown at higher temperature and with hydrogen as carrier gas, every InGaN layer can be grown on a similar surface, which provides the same QD formation parameters for every InGaN layer. In order to protect the InGaN QD structure, a nominal 1.5-nm GaN capping layer with the same temperature was grown on top of the QDs in sample E. In Figure 1, the mentioned nominal 1.5-nm GaN capping layer was grown in period III, whose growth parameters are the same with the growth carrier gas and temperature of InGaN QDs. It serves as a protective layer to maintain three-dimensional nanostructures, which are presented initially. The InGaN QD growth temperature is lower than that of the InGaN QWs, for example, QWs are grown by a “two-temperature” method [32]. As known, the decomposition of InGaN was suppressed in relatively low temperature. In particular, the GaN barrier growth temperature of sample E is lower than that of the QW samples. In conclusion, the influence of increased temperature on QD dimension is not significant in our experiments. We think that it is different from the QW growth parameters which lead to decomposition, due to the relatively low growth temperature during the whole process and the thin GaN protective film layer.

**Figure 7:**
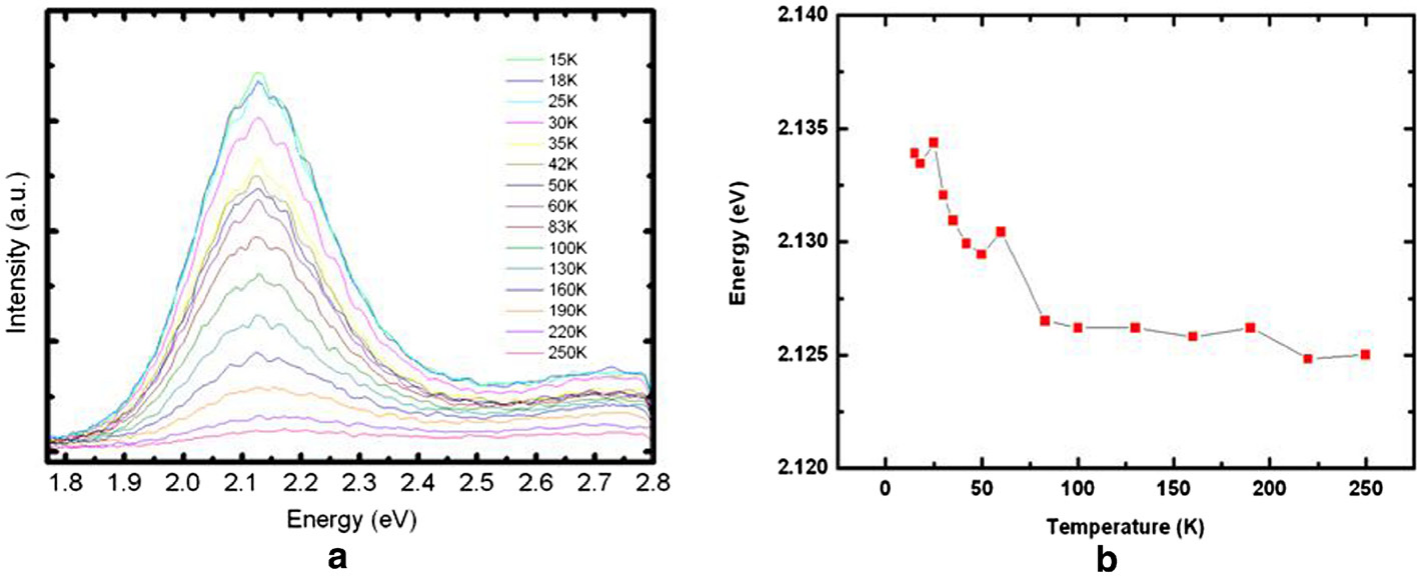
**Temperature-dependent PL spectra. (a)** PL spectra of sample E at different temperatures. **(b)** Temperature-dependent peak energy.

The PL spectra depending on the temperature of sample E are plotted in Figure 7a. Figure 7b summarizes the temperature dependencies of PL peak energy. The redshift of the PL peak from 15 to 250 K is less than 10 meV, while it is more than 30 meV in InGaN bulk or InGaN QW, as reported in references [33,34]. In a previous report about the optical properties of QDs [35], as the temperature increases, carriers can easily thermally transfer into low-energy states from high-energy states. The small redshift observed in our samples may be due to the combined effect of bandgap shift with increasing temperature and carrier redistribution among different QDs. Detailed interpretation of the phenomenon will be left as the topic for future work.

In addition, micro-PL at low temperature shows a sharp peak from single QDs with the same QD growth parameters in our previous reports [16,36]. Based on the micro-PL measurements, we believe that the optical properties of our samples reflect the quantum confinement effect in the InGaN quantum dots.

**Figure 8:**
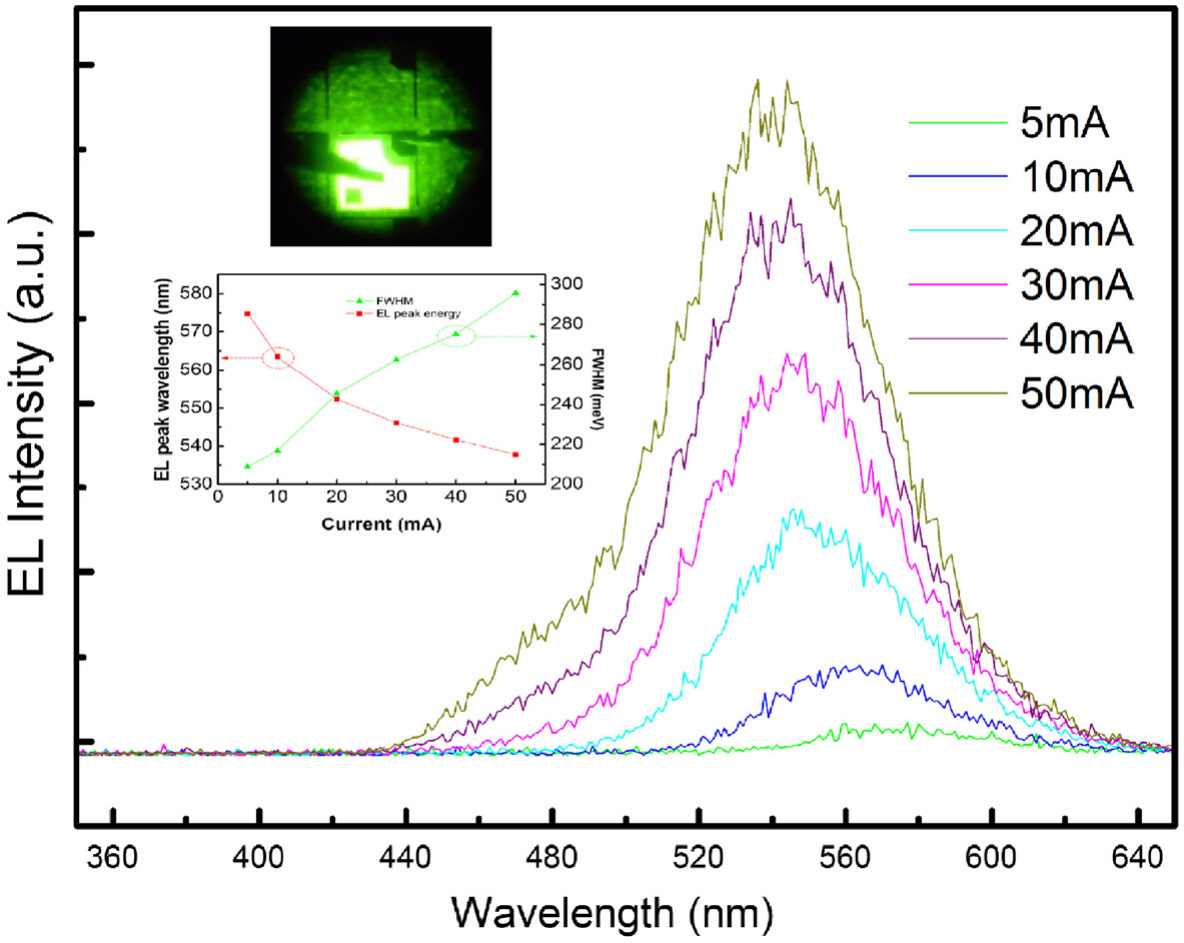
**EL spectra of sample E under different injection currents at room temperature.** The upper inset is the EL image of sample E at 50 mA. The lower inset shows the injection current dependence of the peak wavelength and FWHM of the EL spectra.

The electroluminescence (EL) test results of sample E are shown in Figure 8. As the injection current increases from 5 to 50 mA, the peak wavelength shifts from 574 to 537 nm. The mechanism of EL blueshift may be likely attributed to the carrier screening of QCSE [37]. However, this amount of blueshift is comparable to that of yellow-green LEDs grown on semipolar planes [6]. This means that the QCSE in the QDs is partially suppressed. As the diameter of the QDs is relatively large compared with the height, further reduction of the QCSE can be expected if the aspect ratio becomes larger. The output power is estimated to be 1 mW at a driving current of 50 mA by on-wafer measurements using a calibrated photodiode.

## Conclusions

In summary, by increasing the growth temperature and switching the carrier gas to H_2_, the surface morphology and crystalline quality of GaN barrier layer have been optimized. Compared to the different GaN barrier growth parameters, hydrogen eliminating effect is confirmed by PL results. During the multi-InGaN QD growth, the three-dimensional nanostructure was maintained due to the thin GaN protective layer and relative growth temperature of InGaN QDs and GaN barrier. Based on the optimized growth parameters, a 10-layer InGaN/GaN QD yellow-green LED is successfully grown by MOVPE. The EL wavelength shows a blueshift from 574 to 537 nm as injection current increases from 5 to 50 mA.

## Competing interests

The authors declare that they have no competing interests.

## Authors’ contributions

WL wrote the paper. WL, LW, and YL conceived of and designed the experiments. WL and JW grew the samples. WL, LW, and ZH analyzed the data. WL and JW did all the measurements. All authors discussed the results, contributed to the manuscript text, commented on the manuscript, and read and approved its final version.
